# Variable resolution machine learning optimization of antennas using global sensitivity analysis

**DOI:** 10.1038/s41598-024-77367-w

**Published:** 2024-11-13

**Authors:** Anna Pietrenko-Dabrowska, Slawomir Koziel

**Affiliations:** 1 Faculty of Electronics, Telecommunications and Informatics, Gdansk University of Technology, 80-233 Gdansk, Poland; 2 Engineering Optimization and Modeling Center, Reykjavik University, 101 Reykjavik, Iceland

**Keywords:** Antennas, EM-based design, Multi-resolution analysis, Global optimization, Sensitivity analysis, Surrogate modeling, Nature-inspired algorithms, Engineering, Electrical and electronic engineering

## Abstract

The significance of rigorous optimization techniques in antenna engineering has grown significantly in recent years. For many design tasks, parameter tuning must be conducted globally, presenting a challenge due to associated computational costs. The popular bio-inspired routines often necessitate thousands of merit function calls to converge, generating prohibitive expenses whenever the design process relies on electromagnetic (EM) simulation models. Surrogate-assisted methods offer acceleration, yet constructing reliable metamodels is hindered in higher-dimensional spaces and systems with highly nonlinear characteristics. This work suggests an innovative technique for global antenna optimization embedded within a machine-learning framework. It involves iteratively refined kriging surrogates and particle swarm optimization for generating infill points. The search process operates within a reduced-dimensionality region established through fast global sensitivity analysis. Domain confinement enables the creation of accurate behavioral models using limited training data, resulting in low CPU costs for optimization. Additional savings are realized by employing variable-resolution EM simulations, where low-fidelity models are utilized during the global search stage (including sensitivity analysis), and high-fidelity ones are reserved for final (gradient-based) tuning of antenna parameters. Comprehensive verification demonstrates the consistent performance of the proposed procedure, its superiority over benchmark techniques, and the relevance of the mechanisms embedded into the algorithm for enhancing search process reliability, design quality, and computational efficiency.

## Introduction

Modern antenna design is an intricate task. The reasons are plenty: increasing requirements associated with emerging applications (5G^1,2^, internet of things^3^, microwave imaging^4^, and many others^5–7^), the necessity to provide diverse operating capabilities (e.g., multi-band^8^ and MIMO operation^9^, polarization diversity^10,11^, reconfigurability^12^, beam steering^13^), the need for re-using the same hardware for different operating frequencies^14^, as well as miniaturization trends^15^. The latter has become a particularly important consideration^16–19^, fostering research on small antenna design^20–22^. Fulfilling stringent specifications leads to the development of geometrically sophisticated structures that involve auxiliary components (slots^23^, stubs^24^, transformers^25^, shorting pins^26^), defected ground structures^27^, metamaterials^28^, substrate integrated waveguide cavities^29^, or multi-layer implementations^30^. Topological complexity makes meticulous tuning of antenna parameters imperative; however, it must be realized using electromagnetic (EM) models (equivalent network representations^31,32^ have little or no design utility), and simultaneously applied to all relevant parameters. Further, it might be subject to constraints, especially those related to physical size (e.g., antenna footprint)^33,34^.

Engineering insight combined with parametric studies is still ubiquitous in parameter tuning of antenna structures^35,36^. Nevertheless, rigorous numerical algorithms are the only way to yield optimum designs. Despite the abundance of available methods, EM-based optimization is still challenging due to being CPU-heavy. Even local tuning typically requires dozens to hundreds of EM simulations. Global search is incomparably more expensive^37–39^, particularly when using bio-inspired approaches^40–44^. On the other hand, global optimization is often needed. Examples include tasks featuring multiple local optima (array pattern enhancement^45,46^, frequency selective surface or metasurface design^47,48^), design of compact antennas^49,50^, unavailability of high-quality starting point (e.g., antenna geometry scaling over wide ranges of frequency^51^).

These days, global optimization is predominantly realized using population-based bio-inspired techniques^52–61^ that include genetic and evolutionary algorithms^62,63^, evolutionary strategies^64^, particle swarm optimizers (PSO)^65^, differential evolution (DE)^66^, and many others^67–75^. Arguably, the global search capability results from the exchange of data between the members of population^62^, or mimicking hunting/preying habits^76^ or social behaviour^66^. Unfortunately, nature-inspired methods are tremendously expensive with a few thousands of objective function evaluations on a lower end of typical computational budgets. Such costs are clearly prohibitive for direct EM-driven optimization unless parallelization is possible, contingent upon available resources^77^. Algorithmic speedup has been made possible by incorporating surrogate modeling techniques^78–82^. Practical frameworks are typically iterative procedures, with a fast metamodel (e.g., kriging, neural network, etc.^83–85^) constructed from accumulated EM simulation data and serving as a predictor to identify the optimum design location^86^ (space exploitation), or to produce the infill points targetting the improvement of the model accuracy^87^ (space exploration). These frameworks are often categorized as machine learning (ML) algorithms^88–91^. Despite potential advantages, difficulties in constructing reliable surrogate models, especially in higher-dimensional spaces and over wide ranges of design variables and frequencies, limit the range of applicability of the ML methods^92–94^. A possible workaround is performance-driven modelling techniques^95–98^, incorporation of variable-fidelity EM models^99^, or characteristic point methods^100–102^. The latter leverages reformulation of the problem regarding so-called feature points and close-to-linear dependence of their coordinates on the operating parameters^103^. However, this technique requires that the characteristic points exist over the complete design variable space.

This research introduces a low-cost technique for global antenna optimization, which follows the principles of machine learning. The underlying surrogate model and the core search engine are kriging interpolation and particle swarm optimizer, respectively. The infill criterion employs the merit function improvement according to the prediction of the underlying metamodel. The key acceleration factors encompass dimensionality reduction (realized through fast global sensitivity analysis, FGSA), and variable-resolution EM simulations. FGSA is developed to identify the parameter space directions that maximize antenna response variability. These vectors define the region of interest for the global search stage, executed using low-resolution EM analysis. Subsequent local parameter tuning involves a trust-region gradient based routine operating within the entire parameter space and carried out using high-fidelity EM analysis. Dimensionality reduction and the involvement of low-resolution models translate into superior performance and cost efficiency of our methodology. These have been corroborated by means of comprehensive verification experiments involving four planar antenna structures and several representative benchmark methods. The presented framework consistently yields designs of competitive quality, whereas its average CPU cost amounts to just 140 high-resolution EM analyses per run.

## Variable-resolution machine learning for global antenna optimization using sensitivity-analysis-based dimensionality reduction

This section is devoted to providing the details of the suggested algorithmic framework. We commence by revisiting the formulation of the design task and offering essential background information on variable-resolution EM models (sections “EM-based antenna optimization” and “Variable-resolution computational models”). Subsequently, section “Low-cost global sensitivity analysis” overviews the fast global sensitivity analysis (FGSA), developed to delineate a dimensionality-reduced domain for the machine learning procedure, which is further discussed in section “Global search stage by machine learning”. Additionally, section “Local tuning” delineates the local tuning algorithm, while section “Optimization framework summary” puts together the entire algorithm.

### EM-based antenna optimization

Antenna optimization requires a rigorously defined merit function, which is assumed here to be scalar-valued. If several objectives are present, they are typically combined, e.g., using a weighted function approach^104^ or transformed into constraints^105^. Using the notation and terminology gathered in Table 1, one may define the EM-driven optimization task as

1 $$ {\varvec{x}}^{*} = \arg \;\mathop {\min }\limits_{{{\varvec{x}} \in X}} U({\varvec{x}}) $$

**Table 1:** Parameter adjustment antennas: notation and terminology.

Symbol	Meaning	Comments
***x*** = [*x*_1_ … *x*_*n*_]^*T*^	Designable parameters	Typically, antenna dimensions expressed in mm
*X* = [***l u***]	Parameter space	Parameter space is normally determined using lower and upper bounds on design parameters ***l*** = [*l*_1_ … *l*_*n*_]^*T*^, and ***u*** = [*u*_1_ … *u*_*n*_]^*T*^
*U*(***x***)	Objective (merit) function	The function *U* determines the design quality; it is defined so that better designs correspond to lower values of *U*
*g*_*k*_(***x***) ≤ 0, *k* = 1, …, *n*_*g*_	Inequality constraints	Typically, constraints defined by imposing lower or upper acceptance thresholds for specific antenna responses over selected frequency ranges
*h*_*k*_(***x***) = 0, *k* = 1, …, *n*_*h*_	Equality constraints	Typically, constraints defined by imposing specific target values for selected operating figures (e.g., resonant frequency) of the antenna

In (1), x^*^ represents the optimum design. Antenna responses, necessary to evaluate *U*(***x***) are obtained with the help of full-wave EM simulation. Often, additional constraints are imposed upon (1), cf. the last two rows in Table 1. Their explicit handling, especially if *g*_*k*_ and/or *h*_*k*_ are expensive to evaluate, may be inconvenient. Often, a better option is implicit treatment using a penalty function approach^105^. According to this approach, the problem is reformulated as follows:

2 $$ {\mathbf{x}}^{*} = \arg \mathop {\min }\limits_{{\mathbf{x}}} U_{P} ({\mathbf{x}}) $$

where the objective function *U*_*P*_ is a linear combination of the merit function *U* and the penalty terms

3 $$ U_{P} ({\mathbf{x}}) = U({\mathbf{x}}) + \sum\limits_{k = 1}^{{n_{g} + n_{h} }} {\beta_{k} c_{k} ({\mathbf{x}})} $$

**Table 2:** Representative antenna optimization scenarios.

Design scenario: verbal description	Objective function (1) and constraints	Objective function (3)
Design for best in-band matching within the frequency range *F*	*U*(***x***) = *S*(***x***) = max{*f* ∈ *F* : |*S*_11_(***x***,*f*)|}	*U*_*P*_(***x***) = *U*(***x***)
Design for maximum average in-band gain (in frequency range *F*); ensuring that in-band matching does not exceed − 10 dB in *F*	$U({\mathbf{x}}) = \overline{G}({\mathbf{x}}) = \frac{1}{F}\int\limits_{F} {G({\mathbf{x}},f)df}$ Constraint: $|S_{11} ({\mathbf{x}},f)| \le - 10\;{\text{dB}}\;\;{\text{for}}\;\;f \in F$	$U_{P} ({\mathbf{x}}) = \overline{G}({\mathbf{x}}) + \beta_{1} c_{1} ({\mathbf{x}})^{2}$ where $c_{1} ({\mathbf{x}}) = \left[ {\frac{{\max (S({\mathbf{x}}) + 10,0)}}{10}} \right]^{2}$

Explanation of terms: *f*—frequency, |*S*_11_(***x***,*f*)|—modulus of the reflection coefficient at design ***x*** and frequency *f*, *G*(***x***,*f*)—realized gain.

Therein, the penalty functions *c*_*k*_(***x***), *k* = 1, …, *n*_*g*_ + *n*_*h*_, are defined to quantify constraint violations, whereas coefficients *β*_*k*_ control the contribution of particular penalty terms.

Representative antenna optimization tasks are listed in Table 2. The objective functions are formulated using the implicit approach should any constraints be present. The penalty functions therein (right-hand-side column) are defined to quantify relative constraint violation w.r.t. the given acceptance level (e.g., − 10 dB for |*S*_11_|). The frequency spectrum *F* depends on a particular problem: it may be either a discrete set of target frequencies *F* = {*f*_1_ … *f*_*N*_} for an *N* band antenna or a continuous interval(s), e.g., $F = [f_{1.1} \;f_{1.2} ] \cup [f_{2.1} \;f_{2.2} ] \cup \cdots \cup [f_{N.1} \;f_{N.2} ]$, with *N* being a number of bands.

### Variable-resolution computational models

**Fig. 1:**
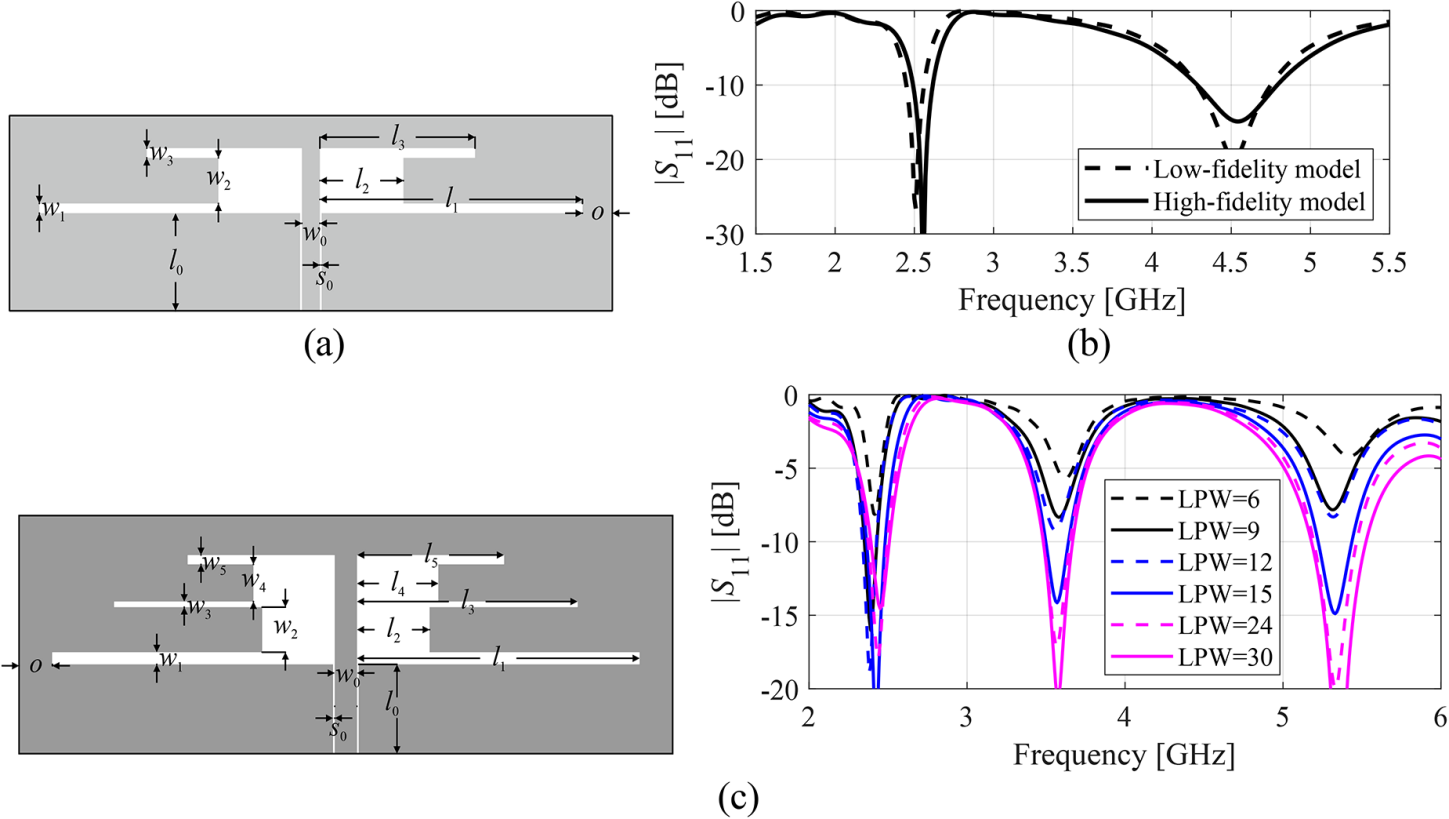
Multi-resolution EM analysis: (**a**) a dual-band antenna; (**b**) |*S*_11_| obtained from the low- (- - -) and high-fidelity EM analysis (—). The high-resolution model simulates in 90 s, whereas the low-fidelity model requires just 25 s of the CPU time; (**c**) model selection: a triple-band antenna and EM models of various fidelities controlled by lines-per-wavelength (LPW) parameter of CST Microwave Studio. Visual inspection indicates that the models with LPW < 15 are heavily distorted and cannot adequately represent antenna characteristics.

Multi-fidelity computational models were applied to accelerate EM-driven procedures for over two decades^106,109–112^. In general, a reduction of the model fidelity can be achieved in diverse ways, for example, through the employment of simplified physics (e.g., equivalent networks in place of full-wave analysis), diminishing the computational domain, ignoring losses, assuming perfect electrical conductors^107^, or reducing the structure’s discretization density^108^. For antennas, the most universal approach is coarse-mesh EM simulation^107^, which is also the only means for the majority of modern antennas. Low-fidelity modelling enables computational speedup while compromising the predictive power, cf. Fig. 1. The acceleration factor is very much dependent on the specific device and may vary from about two to as much as ten, given that the low-resolution EM analysis can render all relevant details of the system response.

We use two models, the low- and high-fidelity ones, ***R***_*c*_(***x***) and ***R***_*f*_(***x***), respectively. ***R***_*f*_(***x***) provides sufficient accuracy to ensure reasonable agreement between EM analysis predictions and the measurements of the antenna prototype. ***R***_*c*_(***x***) is used to conduct global sensitivity analysis, GSA (cf. section “Low-cost global sensitivity analysis”), and carry out global search (cf. section “Global search stage by machine learning”). The final optimization step, i.e., final tuning (cf. section “Local tuning”) will be executed using ***R***_*f*_. Because the two models are well-correlated, there is no need to incorporate any model correction at the GSA stage. Similarly, as the global search step yields an initial design for the final tuning, no low-resolution model correction is required here either. The above properties, directly following the modular architecture of the proposed design framework (cf. section “Optimization framework summary” for the flow diagram), make its implementation considerably simpler as compared to the algorithms relying on model alignment^109,113^.

The selection of the low-fidelity model is carried out through visual inspection of antenna responses as illustrated in Fig. 1c. The mesh density (here, controlled using the lines-per-wavelength, LPW, parameter of CST Microwave Studio) gradually decreased until the response becomes heavily distorted and cannot adequately represent antenna characteristics. For the sample shown in Fig. 1c, this happens for LPW < 15.

### Low-cost global sensitivity analysis

The design optimization framework proposed in this work is contingent upon behavioral models of antenna responses, utilized as predictors at the global search stage. The fundamental difficulty of behavioral modelling is the curse of dimensionality, further aggravated by the sheer size of the design variable space in terms of variable bounds and nonlinearity of antenna frequency characteristics. Dimensionality reduction is imperative to bring down the CPU expenses of surrogate model rendition while ensuring its sufficient accuracy. When it comes to global search procedures, the literature offers several approaches to identify parameters that can be potentially excluded from the search process. These may be categorized into variable screening (e.g., Pearson correlation coefficients^114^, partial correlation coefficients^115^, Morris method^116^), and global sensitivity analysis, GSA (e.g., Sobol indices^117^, regression-based methods^118^, or Jansen method^119^). Unfortunately, the mentioned techniques are generally expensive, i.e., require large numbers of data samples. At the same time, excluding individual variables is rarely an option in antenna design as the electrical and field properties are typically controlled through joint effects of several parameters. Consequently, it is recommended to develop a dimensionality reduction approach that satisfies the following conditions:

- It accounts for the entire parameter space;
- It can be carried out at low computational cost (e.g., less than a hundred of EM analyses);
- It does not focus on identifying individual parameters of high/low importance; instead, it allows for identifying essential parameter space directions that have major influence on antenna response variability.

Below, we provide the details of a fast global sensitivity analysis (FGSA) procedure developed having in mind the above-listed prerequisites.

#### Fast global sensitivity analysis (FGSA)

The main steps of FGSA include allocation of a set of randomly generated parameter vectors, setting up the relocation matrix ***S***, and its spectral analysis leading to the eigenvectors ***e***_*j*_ that represent the parameter space directions having a decreasing effects on the antenna response variability. The corresponding eigenvalues *λ*_*j*_ assess the importance of the particular vectors. By definition, ***e***_*j*_, *j* = 1, …, *n*, constitute an orthonormal basis in the decision variable space *X*. The operating steps of FGSA are as follows:

1. Input parameters:
  Parameter space *X*;
  Computational model ***R***(***x***);
  Number of samples *N*_*s*_;
Generate *N*_*s*_ random vectors ***x***_*s*_^(*k*)^ ∈ *X*, *k* = 1, …, *N*_*s*_, preferably in a uniform manner. Here, we use modified Latin Hypercube Sampling (LHS)^120^;
Acquire EM simulation data ***R***(***x***_*s*_^(*k*)^), *k* = 1, …, *N*_*s*_;
For each *k* = 1, …, *N*_*s*_, find ***x***_*c*_^(*k*)^ = ***x***_*s*_^(*j*min)^ such that
  4 $$ j_{\min } = \arg \mathop {\min }\limits_{\substack{ 1 \le j \le N_{s} \\ j \ne k } } \left\| {{\mathbf{x}}_{s}^{(k)} - {\mathbf{x}}_{s}^{(j)} } \right\| $$
  In other words, ***x***_*c*_^(*k*)^ is the vector closest to ***x***_*s*_^(*k*)^ in the norm sense;
Compute (normalized) relocation vectors
  5 $$ {\mathbf{v}}_{s}^{(k)} = \frac{{{\mathbf{x}}_{c}^{(k)} - {\mathbf{x}}_{s}^{(k)} }}{{\left\| {{\mathbf{x}}_{c}^{(k)} - {\mathbf{x}}_{s}^{(k)} } \right\|}} $$
  and the corresponding (normalized) response variabilities
  6 $$ r_{s}^{(k)} = \frac{{||{\mathbf{R}}({\mathbf{x}}_{c}^{(k)} ) - {\mathbf{R}}({\mathbf{x}}_{s}^{(k)} )||}}{{\left\| {{\mathbf{x}}_{c}^{(k)} - {\mathbf{x}}_{s}^{(k)} } \right\|}} $$
  for *k* = 1, …, *N*_*s*_;
Define a *N*_*s*_ × *n* relocation matrix ***S*** as
  7 $$ {\mathbf{S}} = \left[ {\begin{array}{*{20}c} {r_{s}^{(1)} ({\mathbf{v}}_{s}^{(1)} )^{T} } \\ \vdots \\ {r_{s}^{{(N_{s} )}} ({\mathbf{v}}_{s}^{{(N_{s} )}} )^{T} } \\ \end{array} } \right] $$
  The rows of ***S*** represent relocation vectors normalized with respect to their importance in terms of how they affect antenna response in the norm sense;
Perform spectral analysis of ***S***^121^ in order to find its eigenvectors ***e***_*j*_ (principal components) and the corresponding eigenvalues λ_*j*_, *j* = 1, …, *n*. The eigenvalues are ordered, so that λ_1_ ≥ λ_2_ ≥ … λ_*n*_.

It should be noted that the antenna response variability is computed for the pair of vectors ***x***_*s*_^(*k*)^ and their nearest neighbours ***x***_*c*_^(*k*)^, which is equivalent to computing (large-scale) directional derivatives. The latter determine the changes of frequency characteristics when moving from a given design ***x***_*s*_^(*k*)^ to ***x***_*c*_^(*k*)^. Because the number of such pairs is equal to *N*_*s*_, the overall data gathered this way accounts for typical response sensitivity over the design variable space.

As explained in Step 7, the eigenvalues and eigenvectors are found through a spectral analysis (principal component analysis) of the *N*_*s*_ × *n* relocation matrix ***S***. It is carried out by singular value decomposition of the symmetric *n* × *n* covariance matrix ***C*** = ***S***_*c*_^*T*^***S***_*c*_/(*N*_*c*_ − 1), where ***S***_*c*_ is the centered version of ***S***. We have ***C*** = ***U∑V***^*T*^, where ***U*** is *n* × *n* unitary matrix, ***∑*** is a diagonal matrix containing the eigenvalues, whereas columns of the *n* × *n* matrix ***V*** are the eigenvectors.

A subset of *N*_*d*_ vectors ***e***_*j*_ corresponding to the largest eigenvalues will be used to determine a reduced-dimensionality region of the space *X*, denoted as *X*_*d*_. The set *X*_*d*_ will serve as a domain for the global optimization step, as elaborated on in section “Global search stage by machine learning”.

The number *N*_*d*_ ∈ {1, 2, …, *n*} is found as the smallest integer satisfying

8 $$ \frac{{\sqrt {\sum\nolimits_{j = 1}^{{N_{d} }} {\lambda_{j}^{2} } } }}{{\sqrt {\sum\nolimits_{j = 1}^{n} {\lambda_{j}^{2} } } }} \ge C_{\min } $$

It should be emphasized that the left side of (8) represents an aggregated variability of antenna responses along the first *N*_*d*_ eigenvectors in relation to the total variability; *C*_min_ is a user-defined threshold, set to 0.9 in all verification experiments of section “Algorithm verification”. Selecting *N*_*d*_ as in (8) with *C*_min_ = 0.9 is equivalent to ensuring that the domain-defining directions account for at least ninety percent of the overall response variability.

It should be mentioned that the proposed dimensionality reduction approach may have some resemblance to projection-based model reduction methods such as principal component analysis (PCA) or proper orthogonal decomposition (POD)^134^. At the same time, our approach is focused on explicit identification of the directions responsible for antenna characteristic variability over specific (target) frequency ranges, whereas the computational model itself (here, EM analysis) is considered a black box. In particular, the model is not available in an analytical form and cannot be represented as a parameterized dynamical system. Consequently, techniques such as principal component analysis are not directly applicable to our case and unable to produce corresponding results. On the other hand, PCA is used here as one of the steps of FGSA, specifically spectral analysis of the relocation matrix ***S***. An example of another technique that employs PCA at the optimization stage is^135^, where the authors carry out spectral analysis on the set of designs ranked based on objective function value and obtain a reduced search space this way.

#### Dimensionality reduction: surrogate model domain

As mentioned earlier, the purpose of FGSA is to identify the most influential parameter space directions from the perspective of the system response variability. In particular, the majority of response changes are bounded to the sub-space spanned by the first *N*_*d*_ of these directions (*N*_*d*_ established according to (8)). This region, denoted as *X*_*d*_, will be used as a domain for the global optimization step, and also serve as a surrogate’s domain being the main component of the machine learning (ML) framework of section “Global search stage by machine learning”. Formally, the set *X*_*d*_ is defined as

9 $$ X_{d} = \left\{ {{\mathbf{x}} \in X:{\mathbf{x}} = {\mathbf{x}}_{c} + \sum\limits_{j = 1}^{{N_{d} }} {a_{j} {\mathbf{e}}_{j} } } \right\} \cap X $$

**Fig. 2:**
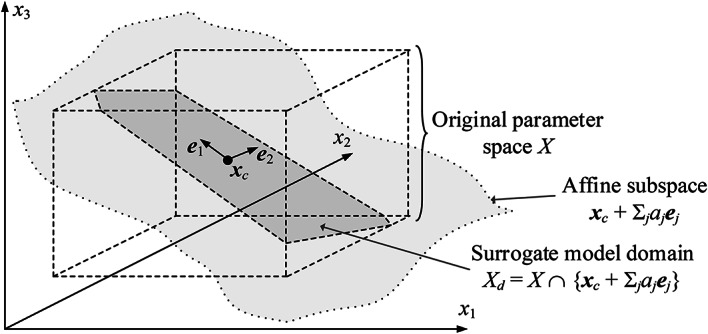
Graphical illustration of the reduced-dimensionality domain *X*_*d*_. Here, the original parameter space is three dimensional, whereas the set *X*_*d*_ is determined using two principal vectors ***e***_1_ and ***e***_2_. Note that *X*_*d*_ is an intersection of *X* and the affine subspace ***x***_*c*_ + Σ_*j*=1,2 _*a*_*j*_***e***_*j*_.

In other words, *X*_*d*_ is an intersection of the original variable space *X* and the affine sub-space.

10 $$ {\varvec{x}}_{c} + a_{{1}} {\varvec{e}}_{{1}} + \ldots + a_{Nd} {\varvec{e}}_{Nd} $$

where ***x***_*c*_ = [***l*** + ***u***]/2 (the centre of *X*), and *a*_*j*_, *j* = 1, …, *N*_*d*_, are real numbers. Figure 2 provides a visualization of the set *X*_*d*_. The main rationale behind dimensionality reduction is to facilitate the construction of the metamodel utilized as a predictor during the machine learning procedure. At the same time, as *X*_*d*_ accounts for the main changes of antenna responses, it provides sufficient flexibility in terms of global optimum identification.

### Global search stage by machine learning

Within the framework proposed in this paper, the initial optimization step is global search conducted in the reduced domain *X*_*d*_. Its role is to identify the region that encapsulates the optimum. The approximate solution found at this stage will be further refined through local tuning (cf. section “Local tuning”). Global search is realized using machine learning (ML). The ML process exploits a kriging interpolation surrogate and the PSO procedure as the core optimization engine. At this stage, we exclusively use the low-fidelity model ***R***_*c*_.

The initial metamodel ***s***^(0)^(***x***) is utilized as a predictor upon launching the machine-learning-based global search algorithm. The assumed modelling technique is kriging interpolation^87^. The model domain is the reduced-dimensionality set *X*_*d*_. The process of establishing ***s***^(0)^(***x***) involves generation of the training datasetm, which is carried out in two steps:

- *N*_*i*_*N*_*d*_ random samples ***x***_*B*_^(*k*)^, *k* = 1, …, *N*_*i*_*N*_*d*_, are allocated uniformly in *X*_*d*_, where *N*_*i*_ is the control parameter (here, we use *N*_*i*_ = 20); the temporary model ***s***_*tmp*_(***x***) is constructed using $\{ {\mathbf{x}}_{B}^{(k)} ,{\mathbf{R}}_{c} ({\mathbf{x}}_{B}^{(k)} )\}_{{k = 1,...,N_{t} N_{d} }}$;
- The infill points are generated by identifying locations corresponding to the maximum mean square error (MSE) of the current surrogate as explained in (11) below.

Model refinement is continued until relative RMS does not exceed *E*_max_ (another control parameter of the proposed framework, here, set to twenty percent), or the total number of samples exceeds 2*N*_*i*_*N*_*d*_ (maximum computational budget). Maximization of MSE enhances the global accuracy of the model within *X*_*d*_. Upon termination, the most recent model ***s***_*tmp*_(***x***) becomes ***s***^(0)^(***x***), which is the first predictor employed in the global search process.

The operating steps of the initial surrogate model construction are as follows:

1. Input parameters:
  Reduced-dimensionality domain *X*_*d*_ (cf. section “Dimensionality reduction: surrogate model domain”);
  Modelling error threshold *E*_max_;
  Initial number of training samples *N*_*i*_*N*_*d*_ (*N*_*i*_ is the user-defined parameter, *N*_*d*_ is the dimensionality of the reduced domain *X*_*d*_).
2. Generate *N*_*i*_*n* samples ***x***_*B*_^(*k*)^ ∈ *X*_*d*_, *k* = 1, …, *N*_*i*_*N*_*d*_, using uniform probability distribution;
3. Set *j* = 0;
4. Evaluate antenna responses ***R***_*c*_(***x***_*B*_^(*k*)^), *k* = 1, …, *N*_*i*_*N*_*d*_ + *j*, using EM simulation;
5. Construct surrogate model ***s***_*tmp*_(***x***) using dataset {***x***_*B*_^(*k*)^,***R***_*c*_(***x***_*B*_^(*k*)^)}_*k* = 1, …, *NiNd* + *j*_;
6. Estimate model error *E*_*tmp*_ using *K*-fold cross-validation^122^, *K* = min{*j*,10};
7. **if** *E*_*tmp*_ < *E*_max_ OR *j* > 2*N*_*i*_*N*_*d*_*.*
        Go to 12;
  **end**
8. Find an infill point by maximizing the mean square error (MSE) of the current
  Surrogate model:
  11 $$ {\mathbf{x}}_{B}^{{(N_{i} N_{d} + j)}} = \arg \mathop {\max }\limits_{{{\mathbf{x}} \in X_{d} }} MSE({\mathbf{s}}_{tmp} ({\mathbf{x}})) $$
9. Set *j* = *j* + 1;
10. Evaluate antenna response ***R***_*c*_(***x***_*B*_^(*NiNd*+*j*)^) using EM simulation;
11. Go to 5;
12. Return ***s***^(0)^(***x***) = ***s***_*tmp*_(***x***);

Having defined ***s***^(0)^, the machine learning (ML) algorithm is launched which iteratively refines the surrogate (using all available EM data), and employs it for producing subsequent approximations of the optimum design. The subsequent models are marked as ***s***^(*j*)^, *j* = 1, 2, … The new candidate design ${\mathbf{x}}_{{}}^{(i + 1)} ,\;i = 0,1,2,...$, is determined by solving

12 $$ {\varvec{x}}^{(i + 1)} = \arg \;\mathop {\min }\limits_{{{\varvec{x}} \in X_{d} }} U_{s} ({\varvec{x}},\;{\varvec{s}}^{(i)} ({\varvec{x}})) $$

The merit function *U*_*S*_ is analytically identical as the one defined in section “EM-based antenna optimization” but evaluated using ***s***^(*i*)^(***x***). This is indicated by the subscript *S* and explicit dependence of *U*_*S*_ on ***s***^(*i*)^.

The underlying search algorithm utilized to solve (12) is the particle swarm optimizer (PSO)^123^. It should be emphasized that any nature-inspired procedure could be used at this stage: due to negligible evaluation cost of *U*_*S*_, the computational budget can be set high, e.g., many thousands of objective function calls, which flattens out the differences between various search procedures (if any). PSO has been chosen due to being representative for this class of methods and widely popular.

From the ML perspective, the problem (12) establishes surrogate-predicted merit function enhancement to be an infill criterion^124^. The model ***s***^(*i*)^(***x***) is built based on all EM simulation data acquired until iteration *i* inclusive, i.e., $\{ {\mathbf{x}}_{B}^{(k)} ,{\mathbf{R}}({\mathbf{x}}_{B}^{(k)} )\}_{{k = 1,...,2N_{i} N_{d} + i}}$. Therein, ${\mathbf{x}}_{B}^{{(2N_{i} N_{d} + i)}} = {\mathbf{x}}^{(i)} \;{\text{for}}\;i = 1,2,...$. The global search stage is considered accomplished if $||{\mathbf{x}}^{(i + 1)} - {\mathbf{x}}^{(i)} || < \varepsilon$ or the EM-evaluated objective function did not improve during *N*_*no_improve*_ most recent iterations. In the verification part of the work (section “Algorithm verification”), the following values of the mentioned parameters will be used: *ε* = 10^−2^ and *N*_*no_improve*_ = 20.

It should be noted that initial surrogate model construction and global search stage use different infill criteria, which is MSE maximization for initial model rendition, and objective function improvement for the global search stage. The rationale behind it is as follows. We aim at the initial surrogate model being as accurate as possible (therefore MSE is used as an infill criterion) so that global search. Having better model will expedite further optimization steps. Also, an acceptance threshold is introduced (*E*_max_) achieving of which terminates the model construction process. Consequently, the used arrangement allows for a better control of the model accuracy. In the next stage, our only concern is identification of the region containing global optimum, therefore, objective function improvement as an infill criterion seems more appropriate keeping in mind computational efficiency (the process is fast anyhow due to initiating it with already good surrogate). Furthermore, the global search process is followed by local tuning, which allows for more precise optimum allocation. At the same time, it should be mentioned that many ML frameworks use different infill criteria, typically to ensure more balanced between exploration and exploitation (e.g., expected improvement)^124^.

### Local tuning

**Fig. 3:**
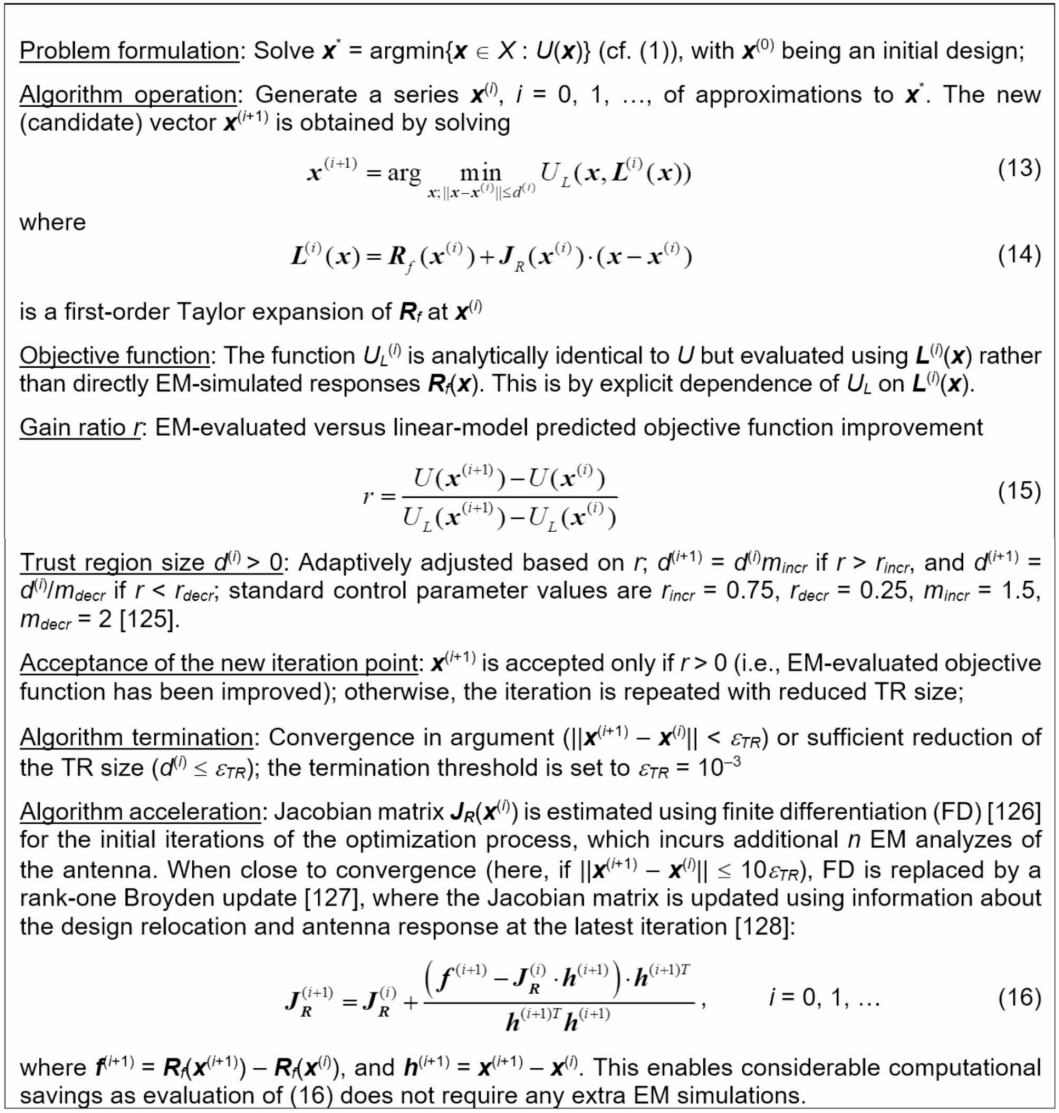
Accelerated trust-region (TR) search procedure employing a rank-one Broyden sensitivity updating formula^128^.

The ML-based global search is executed in the restricted domain *X*_*d*_, which may prevent the process from identifying a truly optimum design. Therefore, a supplementary local tuning is employed, which is carried out over the entire parameter space *X* using accelerated gradient-based optimization. We use the trust-region (TR) routine^125^ characterized in Fig. 3. The TR procedure employs a linear model of antenna outputs as a predictor, and generates subsequent approximations of the optimum design by optimizing the predictor-based merit function over the search region of adaptively adjusted radius. When the algorithm is close to convergence, specifically, when ||***x***^(*i*+1)^ − ***x***^(*i*)^|| < 10*ε*_*TR*_, the sensitivity updating scheme is changed from full finite differentiation^126^, to rank-one Broyden updating formula^127^. This reduces the number of EM simulations from *n* + 1 to one per iteration. This substitution results in a notable enhancement of the computational efficiency. To ensure dependability, local tuning is executed using the high-fidelity model ***R***_*f*_.

### Optimization framework summary

The proposed variable-resolution framework for global antenna optimization puts together the algorithmic mechanisms described in section “Low-cost global sensitivity analysis” through “Local tuning”. Among these, the fast global sensitivity analysis (FGSA) and surrogate-assisted ML search are conducted using the low-fidelity EM model ***R***_*c*_ (cf. section “Variable-resolution computational models”). Only the final (local) parameter tuning is realized using the high-resolution model ***R***_*f*_.

Table 3 enumerates the framework’s control parameters. The last three parameters (*ε*, *N*_*no_improve*_, *ε*_*TR*_) are related to the termination conditions, i.e., they only affect the optimization process precision. The remaining ones, *N*_*r*_, *N*_*i*_, and *E*_max_, determine the reliability of the sensitivity analysis and intended accuracy of the metamodel. Consequently, their values are not critical because the number of random observables used by FGSA does not affect the outcome significantly. In contrast, *N*_*i*_ and *E*_max_ are only used to construct the initial metamodel, which is further enhanced during the ML process. Based on these comments, the proposed algorithm does not need to be specifically adjusted for a specific problem. This is illustrated by using an identical setup (cf. the last column of Table 3) for all numerical experiments of section “Algorithm verification”.

**Table 3:** Control parameters of the proposed variable-resolution global optimization algorithm.

Parameter	Meaning	Default value
*N*_*r*_	Number of random observables for fast global sensitivity analysis (FGSA), cf. section “Low-cost global sensitivity analysis”	50
*N*_*i*_	Multiplier for the number of uniformly-distributed data samples for initial surrogate model construction; the actual number of samples is *N*_*i*_*N*_*d*_, with *N*_*d*_ being the dimensionality of the reduced domain *X*_*d*_ (cf. section “Global search stage by machine learning”)	20
*E*_max_	Maximum value of relative RMS error of the initial surrogate model (error estimated using cross-validation), cf. section “Global search stage by machine learning”	20%
*ε*	Termination threshold for convergence in argument, cf. section “Global search stage by machine learning”	10^−2^
*N*_*no_improve*_	Termination threshold for no objective function value improvement, cf. section “Global search stage by machine learning”	10
*ε*_*TR*_	Termination threshold for local parameter tuning stage, cf. section “Local tuning”	10^−3^

**Fig. 4:**
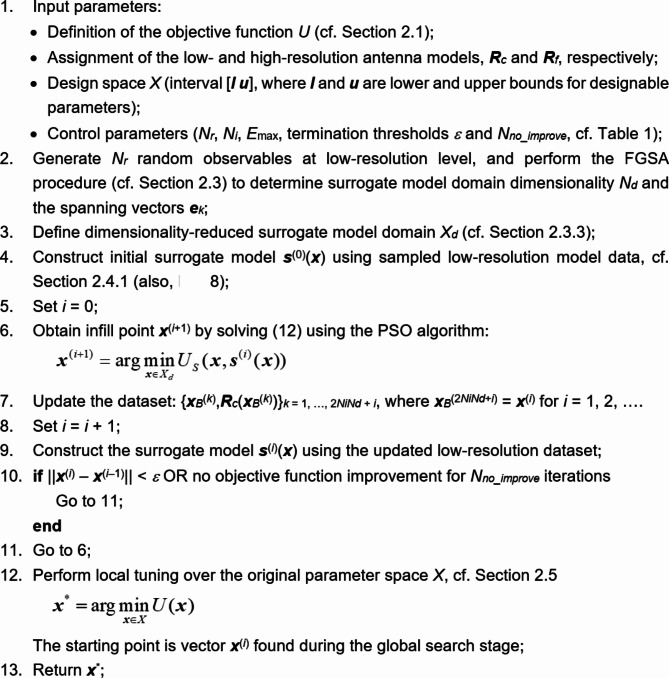
Pseudocode of the variable-resolution machine-learning search procedure. The essential part of the algorithm is dimensionality-reduced surrogate whose domain is established using the proposed fast global sensitivity analysis scheme.

**Fig. 5:**
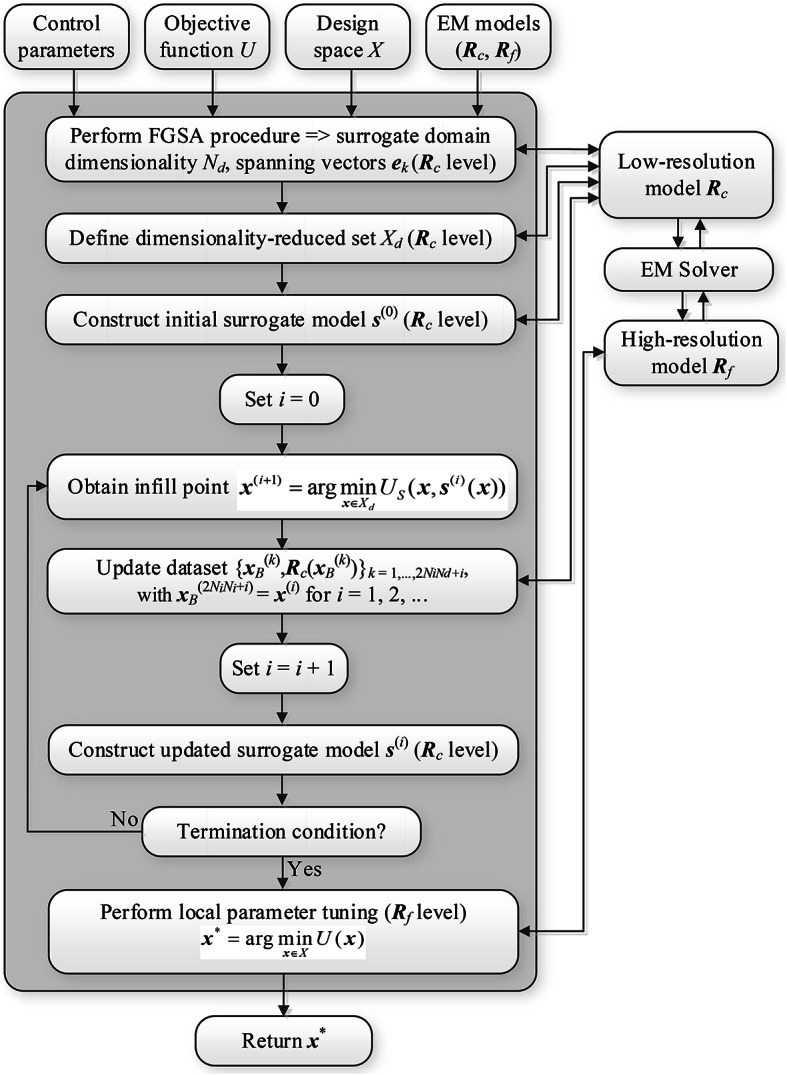
Flow diagram of the variable-resolution machine-learning procedure for global antenna optimization.

Figure 4 illustrates the pseudocode of our optimization framework. The flow diagram is presented in Fig. 5. The majority of the search process, i.e., FGSA (Step 2), dimensionality-reduced domain definition (Step 3), construction of the initial surrogate (Step 4), as well as ML-based search (Steps 6 through 10), is performed using the low-fidelity model ***R***_*c*_. Only the final (gradient-based) parameter tuning is realized with the help of the high-fidelity model ***R***_*f*_ to secure optimization process dependability.

## Algorithm verification

We begin by illustrating the FGSA process in section “FGSA examples”. The test cases are outlined in section “Verification antenna structures”. For verification, our procedure is compared in section “Results” to several benchmark methods representing bio-inspired optimizers, machine learning, and conventional gradient-governed search. The latter is included to corroborate the presence of multiple local optima for the considered problems. Due to the stochastic nature of the algorithms, they are executed ten times each. The outcomes obtained this way are investigated in section “Discussion”.

### FGSA examples

**Fig. 6:**
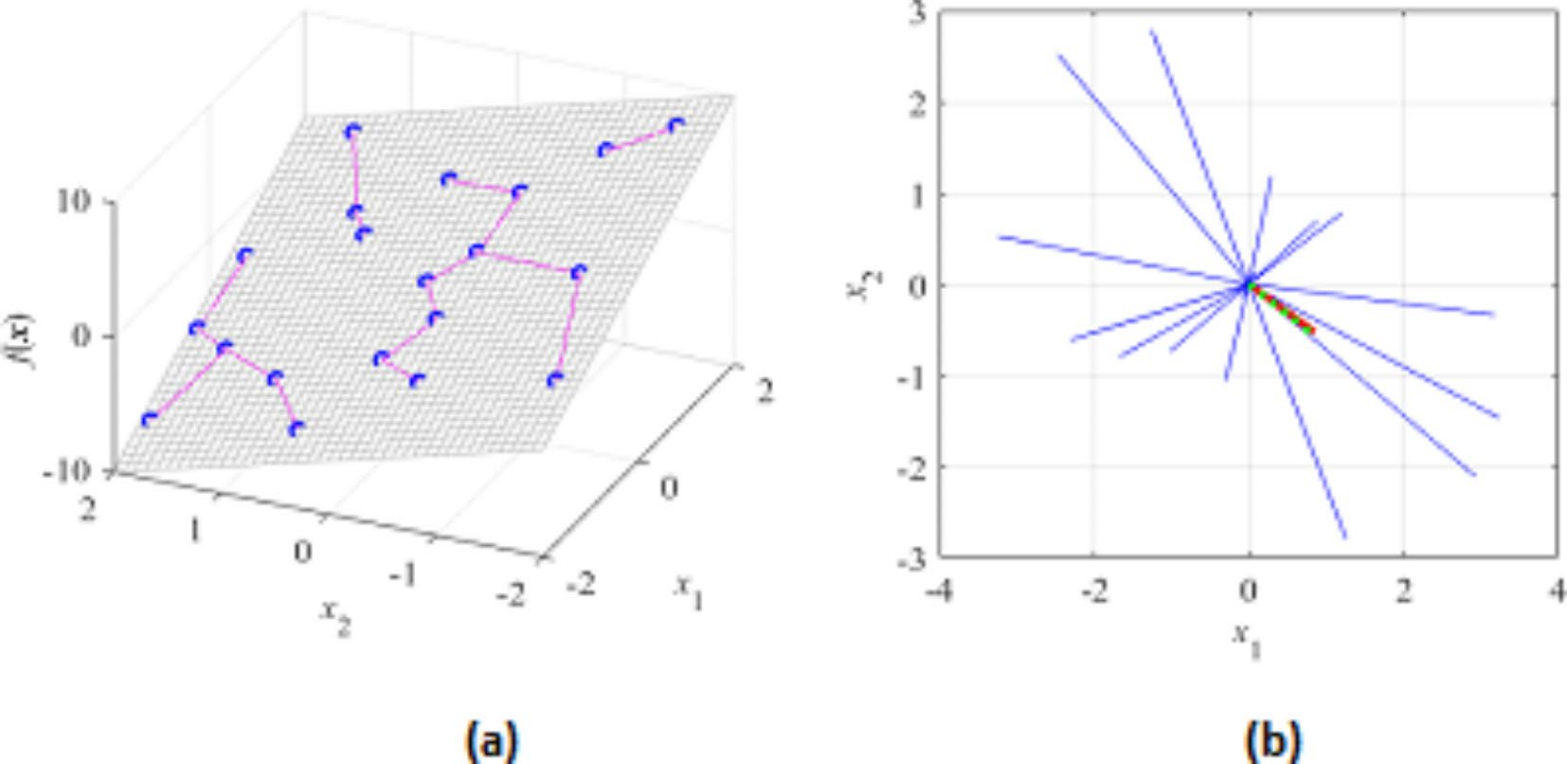
FGSA illustration using a linear function *f*(***x***) = *f*([*x*_1_ *x*_2_]^*T*^) = 3*x*_1_ − 2*x*_2_: (**a**) the surface plot of the function (gray), twenty random observables ***x***_*s*_^(*k*)^ (circles), and relocation vectors ***x***_*c*_^(*k*)^ − ***x***_*s*_^(*k*)^ (line segments); (**b**) relocation matrix vectors *r*_*s*_^(*k*)^***v***_*s*_^(*k*)^ (thin lines), the largest principal component ***e***_1_ (thick solid line), and the normalized gradient ***g*** = [3 − 2]^*T*^/13^1/2^ (thick dotted line). In this example, all function variability occurs along the gradient ***g*** (the function is constant in the direction orthogonal to ***g***), which is well aligned with the vector ***e***_1_, obtained using the proposed FGSA.

**Fig. 7:**
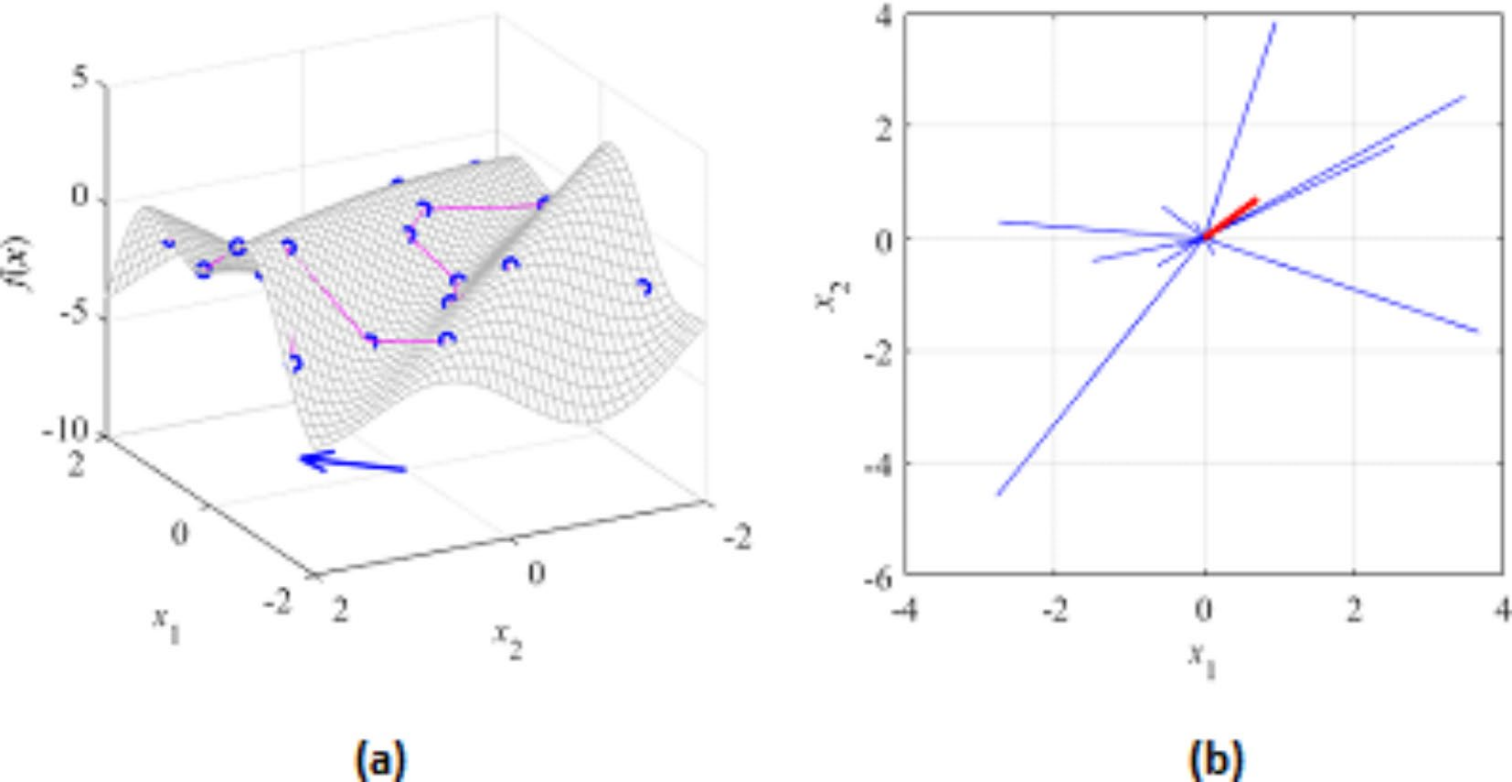
FGSA illustration using a nonlinear function of two variables: (**a**) surface plot of the first function (gray), twenty random observables ***x***_*s*_^(*k*)^ (circles), and relocation vectors ***x***_*c*_^(*k*)^ − ***x***_*s*_^(*k*)^ (line segments), as well as the principal component ***e***_1_ (thick arrow); (**b**) relocation matrix vectors *r*_*s*_^(*k*)^***v***_*s*_^(*k*)^ (thin lines), and the largest principal component ***e***_1_ (thick solid line). It can be noticed that the vector ***e***_1_ obtained using FGSA visually corresponds to the direction of the largest variability of the function *f*(***x***).

Here, we consider two examples illustrating the FGSA procedure of section “Fast global sensitivity analysis (FGSA)”. We start with a simple linear function *f*(***x***) = *f*([*x*_1_ *x*_2_]^*T*^) = 3*x*_1_ − 2*x*_2_, shown in Fig. 6. FGSA has been executed using twenty random samples. The first eigenvector ***e***_1_ = [0.84 − 0.53]^*T*^, which agrees with the normalized gradient of *f*(***x***), ***g*** = [0.83 − 0.55]^*T*^ (the latter determines the direction of maximum variability owing to linearity of *f*). Figure 7 shows another example, where the function *f* is defined to allow visual assessment of the maximum variability (the vector perpendicular to the ‘ripples’). Again, this observation agrees with the results obtained using FGSA.

### Verification antenna structures

**Fig. 8:**
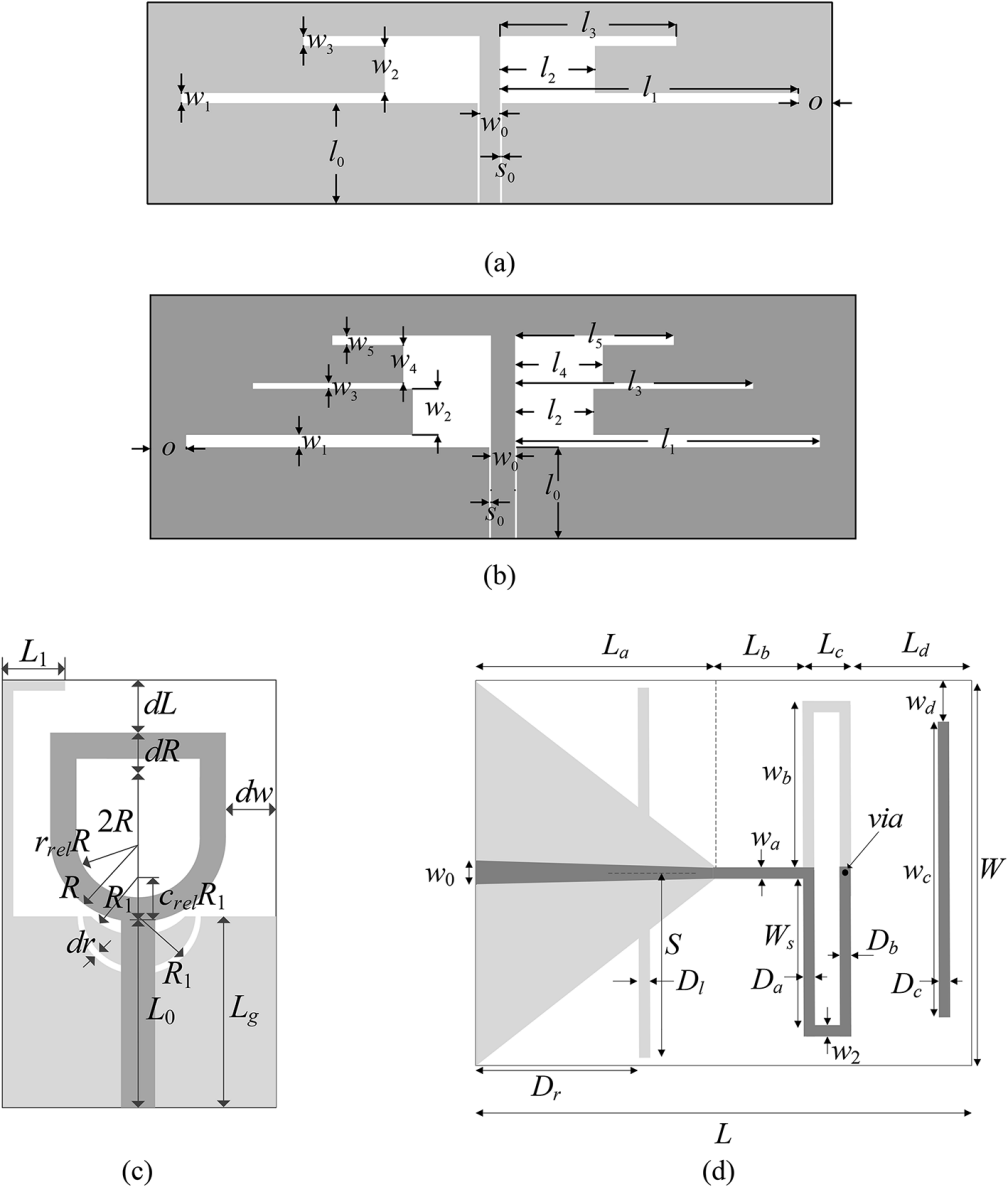
Test devices: (**a**) Antenna I^129^, (**b**) Antenna II^130^, (**c**) Antenna III^131^, (d) Antenna IV^132^. The light-gray shade is used to indicate the ground-plane metallization.

The geometries of test antennas have been shown in Fig. 8. Table 4 contains the essential data on all structures (substrate parameters, design variables, target frequencies, parameter ranges, etc.). The devices are named Antenna I, II, III, and IV, respectively. The EM models are prepared and evaluated in CST Microwave Studio. The time-domain solver is utilized for simulation purposes^133^. The low-resolution models ***R***_*c*_ are set up by restricting the mesh density of the respective devices so that the simulated response still properly represents the critical features of the antenna, such as resonances. The high-resolution model ***R***_*f*_ is determined using the grid convergence study with the discretization set to the level increasing of which does not have visible effects on frequency characteristics. The grid convergence study is understood as gradual increase of the mesh density and visual inspection of the antenna characteristics produced by EM simulation. The high-fidelity model is set up at the mesh density for which its further refinement does not alter the antenna responses in a noticeable manner. The time evaluation ratio between ***R***_*f*_ and ***R***_*c*_ is 3.7, 2.3, 8.3, and 3.8 for Antenna I through IV. Clearly, higher ratio will lead to higher computational savings.

**Table 4:** Verification antenna structures.

Parameter	Antenna structure			
	Antenna I	Antenna II	Antenna III	Antenna IV
Substrate	RO4350 (*ε*_*r*_ = 3.5, *h* = 0.76 mm)	RO4350 (*ε*_*r*_ = 3.5, *h* = 0.76 mm)	RF-35 (*ε*_*r*_ = 3.5, *h* = 0.762 mm)	RO4003 (*ε*_*r*_ = 3.38, *h* = 1.5 mm)
Design parameters^$^	***x*** = [*l*_1_ *l*_2_ *l*_3_ *w*_1_ *w*_2_ *w*_3_]^*T*^	***x*** = [*l*_1_ *l*_2_ *l*_3*r*_* l*_4_ *l*_5*r*_* w*_1_ *w*_2_ *w*_3_ *w*_4_ *w*_5_]^*T*^	***x*** = [*L*_0_ *dR R r*_*rel*_* dL dw Lg L*_1_ *R*_1_ *dr c*_*rel*_]^*T*^	***x*** = [*L*_*a*_* L*_*b*_* L*_*c*_* L*_*d*_* W w*_*a*_* D*_*a*_* D*_*b*_* D*_*c*_* D*_*lr*_* D*_*rr*_* S*_*r*_* w*_*br*_* w*_*cr*_]^*T*^
Other parameters^$^	*l*_0_ = 30, *w*_0_ = 3, *s*_0_ = 0.15, *o* = 5	*l*_3_ = *l*_3*r*_*l*_1_ and *l*_5_ = *l*_5*r*_*l*_3_; *l*_0_ = 30, *w*_0_ = 3, *s*_0_ = 0.15, *o* = 5	*w*_0_ = 1.7	*D*_*l*_ = *D*_*lr*_*L*_*a*_, *D*_*r*_ = *D*_*rr*_*L*_*a*_, *S* = *S*_*r*_*W*, *w*_*b*_ = *w*_*br*_*W*/2, *w*_*c*_ = *w*_*cr*_*W*, *w*_0_ = 3.4
EM model	CST Microwave Studio	CST Microwave Studio	CST Microwave Studio	CST Microwave Studio
***R***_*c*_ (low-resolution model)	~ 60,000 mesh cells Simulation time 25 s	~ 71,000 mesh cells Simulation time 35 s	~ 210,000 mesh cells Simulation time 51 s	~ 81,000 mesh cells Simulation time 39 s
***R***_*f*_ (high-resolution model)	~ 410,000 mesh cells Simulation time 92 s	~ 270,000 mesh cells Simulation time 80 s	~ 2,300,000 mesh cells Simulation time 424 s	~ 550,000 mesh cells Simulation time 150 s
Target operating frequencies [GHz]	2.45 GHz 5.3 GHz	2.45 GHz 3.6 GHz 5.3 GHz	3.1 GHz to 10.6 GHz	2.5 GHz
Design goals	Minimize reflection at all operating frequencies	Minimize reflection at all operating frequencies	Minimize reflection within the entire UWB band	Maximize realized gain in ± 100 MHz bandwidth centred at ***f***_*t*_; Constraint: |*S*_11_|≤ − 10 dB at the same bandwidth
Parameter space *X*	***l*** = [15 3 0.35 0.2 1.8 0.5]^*T*^ ***u*** = [50 12 0.85 1.5 4.3 2.7]^*T*^	***l*** = [20 3 0.6 3 0.6 0.2 0.2 0.2 0.2 0.2]^*T*^ ***u*** = [50 5 0.85 5 0.85 2.2 4.2 2.2 4.2 2.2]^*T*^	***l*** = [4.0 0.0 3.0 0.1 0.0 0.0 4.0 0.0 2.0 0.2 0.2]^*T*^ ***u*** = [15.0 6.0 8.0 0.9 5.0 8.0 15.0 6.0 5.0 1.0 0.9]^*T*^	***l*** = [15 5 1 15 25 0.5 1 1.5 1.5 0.05 0.4 0.5 0.5 0.5]^*T*^ ***u*** = [35 25 8 40 60 2.5 3.0 4.5 4.5 0.25 0.9 1.0 1.0 1.0]^*T*^

^$^Dimensions in mm, except relative one (with subscript *r*), which are unitless.

All verification problems are intrinsic because of large parameter spaces (dimensionality from six to fourteen, the mean value of the ratio between the upper and lower bound reaching 4.2, 8.4, 2.8, and 2.6 for Antennas I through IV) as well as considerable nonlinearity of antenna characteristics, both with respect to frequency and design variables.

### Results

Antennas I through IV were optimized using the algorithm of section “Variable-resolution machine learning for global antenna optimization using sensitivity-analysis-based dimensionality reduction”, and several comparison methods listed in Table 5. For all antenna structures, the proposed procedure is executed using identical control parameter setup as elaborated on in Table 3. The benchmark set includes four algorithms:

- Algorithm I: particle swarm optimizer (PSO)^123^, employed as a flagship bio-inspired method. It is executed with the budget of 500 (Version I) and 1000 (Version II) objective function calls. These numbers are low for population-based methods; however, the algorithm running time (two to three days) is considerable due to repetitive EM simulations.
- Algorithm II: differential evolution utilizes as one of the most popular state-of-the-art population based algorithms^136^. The algorithm is excuted using 1000 function calls as a computational budget.
- Algorithm III: Grey Wolf optimization^37^, one of the recent and popular bio-inspired algorithms. Again, the computational budget is set to 1000 EM simulations.
- Algorithm IV: multiple-start gradient search, utilized to corroborate multimodality of the considered optimization tasks. In this study, it is conducted by means of the TR algorithm similar to the one explained in Fig. 4.
- Algorithm V: a machine-learning procedure working with kriging surrogates and the same mechanism for generating the infill points as the proposed technique. The major difference is that the algorithm operates within the original design space *X*. This technique is considered to demonstrate the advantages of dimensionality reduction.
- Algorithm VI: a machine-learning routine working along the lines of what was considered in section “Variable-resolution machine learning for global antenna optimization using sensitivity-analysis-based dimensionality reduction” but exclusively using the ***R***_*f*_ model. We include this method to quantify the advantages of employing variable-resolution EM simulations.

**Table 5:** Benchmark algorithms.

Method	Algorithm type	Setup
This work	FGSA-based surrogate-assisted machine-learning framework with dimensionality reduction and variable-resolution EM models	Control parameters: *N*_*r*_ = 50, *N*_*i*_ = 20, *E*_max_ = 20%, *ε* = 10^–2^, *N*_*no_improve*_ = 20, *ε*_*TR*_ = 10^–3^ (see Table 1 for an explanation of terms)
I	Particle swarm optimizer (PSO)	Swarm size *N* = 10, standard control parameters (*χ* = 0.73, *c*_1_ = *c*_2_ = 2.05); the number of iterations set to 50 (version I) and 100 (version II)
II	Differential evolution (DE) ^135^	Algorithm setup: • Population size *N* = 10; • Standard control parameters (crossover probability *CR* = 0.9, differential weight *F* = 0.8); Number of iterations set to 100
III	Gray Wolf optimizer (GWO)^37^	Algorithm setup: • Population size *N* = 10; • Standard control parameters, cf.^37^; Number of iterations set to 100
IV	Trust-region gradient-based optimizer^125^	Random initial design, response gradients estimated using finite differentiation, termination criteria based on convergence in argument and reduction of the trust region size^125^
V	Machine-learning procedure	Algorithm setup: • Initial surrogate set up to ensure relative RMS error not higher than 20% with the maximum number of training samples equal to 400; • Algorithm operates in the original parameter space (no dimensionality reduction); • Infill criterion: minimization of the predicted objective function
VI	Machine-learning procedure	Algorithm setup: • The method is the same as the proposed one; however, the algorithm operates at the level of high-resolution EM models; • Control parameters: default values as in Table 1

Tables 6, 7, 8, 9 contain the results for all test antennas. Due to the presence of random components within the algorithms, they were executed ten times each. The reported data include the cost function value and the CPU cost entailed by the search process, both averaged over all algorithm runs. Additionally, the tables provide information about the number of runs for which a given procedure was able to produce design whose actual operating frequencies match the assumed targets. The success rate can be considered a measure of the reliability and solution repeatability of the search process.

**Table 6:** Antenna I: optimization results.

Optimization algorithm	Performance figure		
	Average objective function value [dB]	Computational cost^$^	Success rate^#^
Algorithm I: PSO (50 iterations)	− 18.2	500	9/10
Algorithm I: PSO (100 iterations)	− 19.3	1000	10/10
Algorithm II: DE (100 iterations)	− 19.8	1000	9/10
Algorithm III: GWO (100 iterations)	− 19.1	1000	9/10
Algorithm IV: Trust-region gradient-based algorithm	− 13.5	84.2	6/10
Algorithm V: Machine learning operating in the original parameter space *X*	− 20.7	457.8	10/10
Algorithm VI: FSGA-based machine learning operating at the level of high-resolution model only	− 20.6	221.8	10/10
Proposed algorithm	− 24.5	104.8	10/10

^$^The cost expressed in terms of the number of EM simulations of the antenna structure under design.

^#^Number of algorithms runs at which the operating frequencies were allocated in the vicinity of the target frequencies.

**Table 7:** Antenna II: optimization results.

Optimization algorithm	Performance figure		
	Average objective function value [dB]	Computational cost^$^	Success rate^#^
Algorithm I: PSO (50 iterations)	− 10.8	500	5/10
Algorithm I: PSO (100 iterations)	− 13.8	1000	8/10
Algorithm II: DE (100 iterations)	− 12.5	1000	8/10
Algorithm III: GWO (100 iterations)	− 11.3	1000	7/10
Algorithm IV: Trust-region gradient-based algorithm	− 7.8	105.8	4/10
Algorithm V: Machine learning operating in the original parameter space *X*	− 13.5	470.0	10/10
Algorithm VI: FSGA-based machine learning operating at the level of high-resolution model only	− 15.4	303.7	10/10
Proposed algorithm	− 17.7	192.3	10/10

^$^The cost expressed in terms of the number of EM simulations of the antenna structure under design.

^#^Number of algorithms runs at which the operating frequencies were allocated in the vicinity of the target frequencies.

**Table 8:** Antenna III: optimization results.

Optimization algorithm	Performance figure		
	Average objective function value [dB]	Computational cost^$^	Success rate^#^
Algorithm I: PSO (50 iterations)	− 12.3	500	9/10
Algorithm I: PSO (100 iterations)	− 12.6	1000	10/10
Algorithm II: DE (100 iterations)	− 12.7	1000	10/10
Algorithm III: GWO (100 iterations)	− 11.9	1000	9/10
Algorithm IV: Trust-region gradient-based algorithm	− 7.8	99.2	5/10
Algorithm V: Machine learning operating in the original parameter space *X*	− 11.8	471.6	9/10
Algorithm VI: FSGA-based machine learning operating at the level of high-resolution model only	− 13.2	308.1	10/10
Proposed algorithm	− 13.1	79.8	10/10

^$^The cost expressed in terms of the number of EM simulations of the antenna structure under design.

^#^Number of algorithms runs at which the maximum in-band matching was reduced below − 10 dB.

**Table 9:** Antenna IV: optimization results.

Optimization algorithm	Performance figure		
	Average objective function value [dB]^&^	Computational cost^$^	Success rate^#^
Algorithm I: PSO (50 iterations)	6.1	500	9/10
Algorithm I: PSO (100 iterations)	6.8	1000	10/10
Algorithm II: DE (100 iterations)	7.0	1000	10/10
Algorithm III: GWO (100 iterations)	6.8	1000	10.10
Algorithm IV: Trust-region gradient-based algorithm	− 1.1	144.3	1/10
Algorithm V: Machine learning operating in the original parameter space *X*	7.9	583.3	10/10
Algorithm VI: FSGA-based machine learning operating at the level of high-resolution model only	8.0	370.4	10/10
Proposed algorithm	8.1	173.3	10/10

^&^The values reported in the table refer to the realized gain at the target operating frequency of 2.5 GHz.

^$^The cost expressed in terms of the number of EM simulations of the antenna structure under design.

^#^Number of algorithms runs at which the operating frequencies were allocated in the vicinity of the target frequency.

**Fig. 9:**
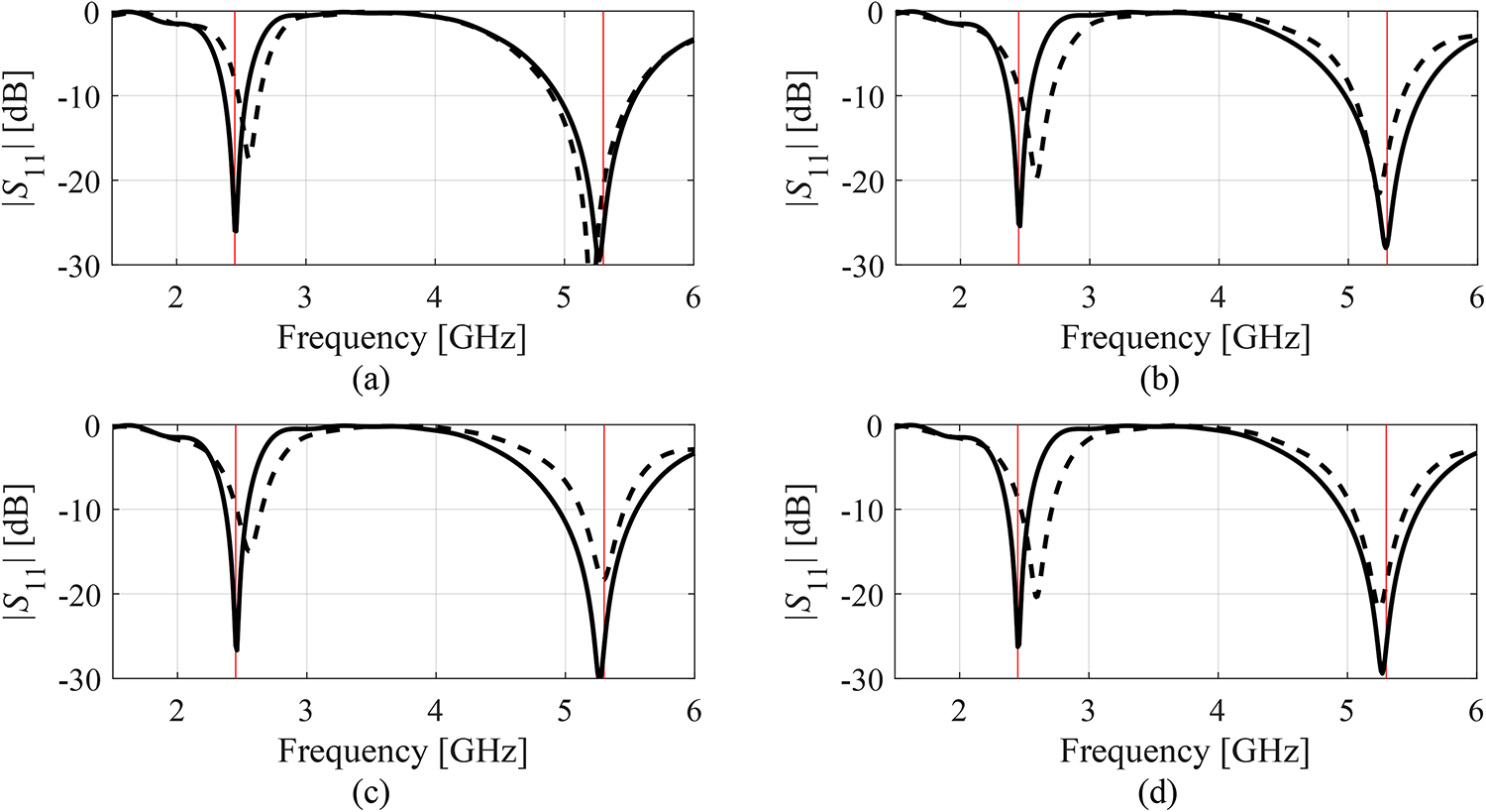
|*S*_11_| of Antenna I at the designs produced by the proposed variable-resolution surrogate-assisted machine learning framework. Shown is the data for selected algorithm runs (plots (**a**) through (**d**)). The design ***x***^(0)^ generated by the global optimization step (- - -) and the final design (—). Target operating frequencies marked with vertical lines.

**Fig. 10:**
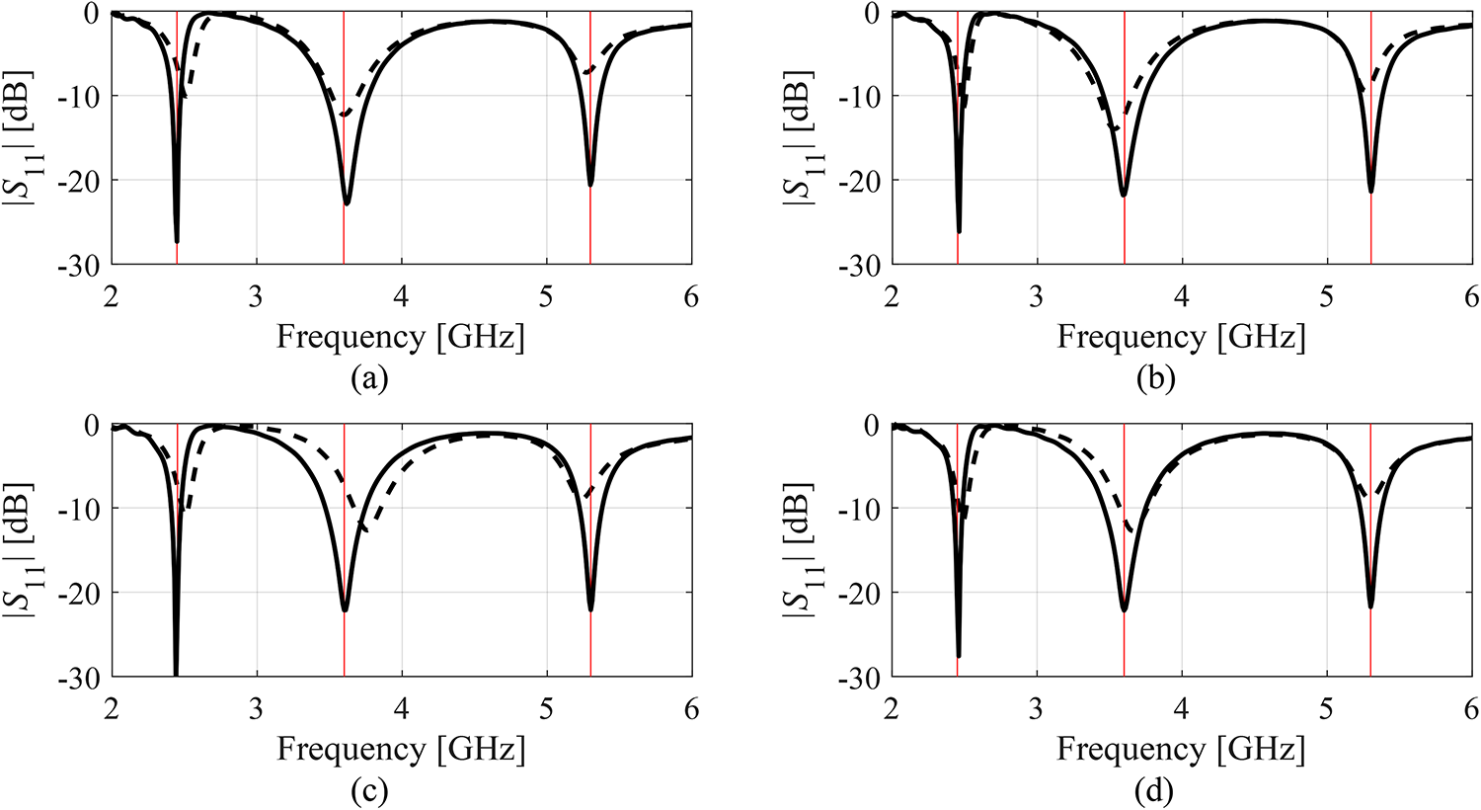
|*S*_11_| of Antenna II at the designs produced by the proposed variable-resolution surrogate-assisted machine learning framework. Shown is the data for selected algorithm runs (plots (**a**) through (**d**)). The design ***x***^(0)^ generated by the global optimization step (- - -) and the final design (—). Target operating frequencies marked with vertical lines.

**Fig. 11:**
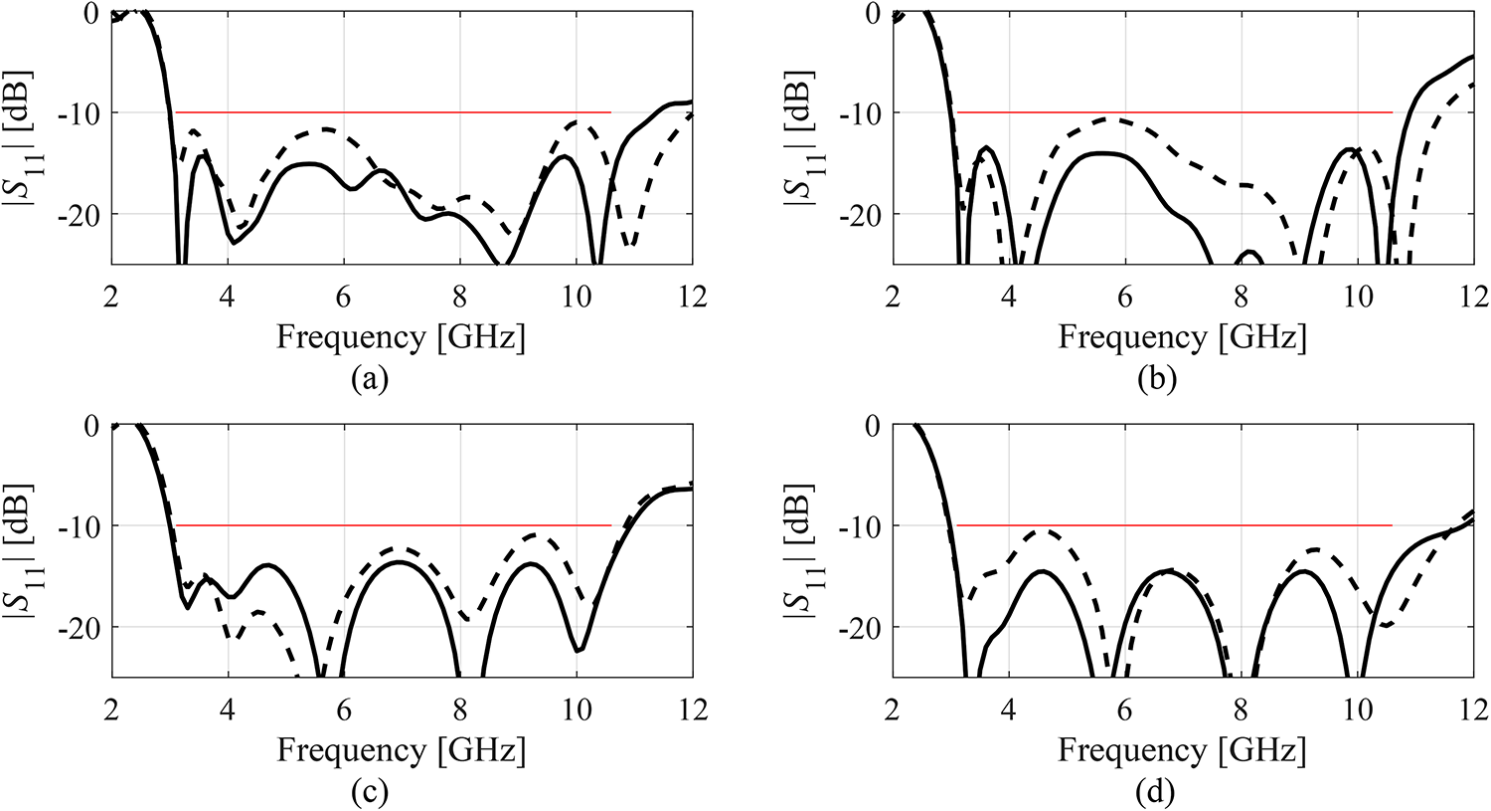
|*S*_11_| of Antenna III at the designs produced by the proposed variable-resolution surrogate-assisted machine learning framework. Shown is the data for selected algorithm runs (plots (**a**) through (**d**)). The design ***x***^(0)^ generated by the global optimization step (- - -) and the final design (—). Target operating bandwidth marked with the horizontal line.

**Fig. 12:**
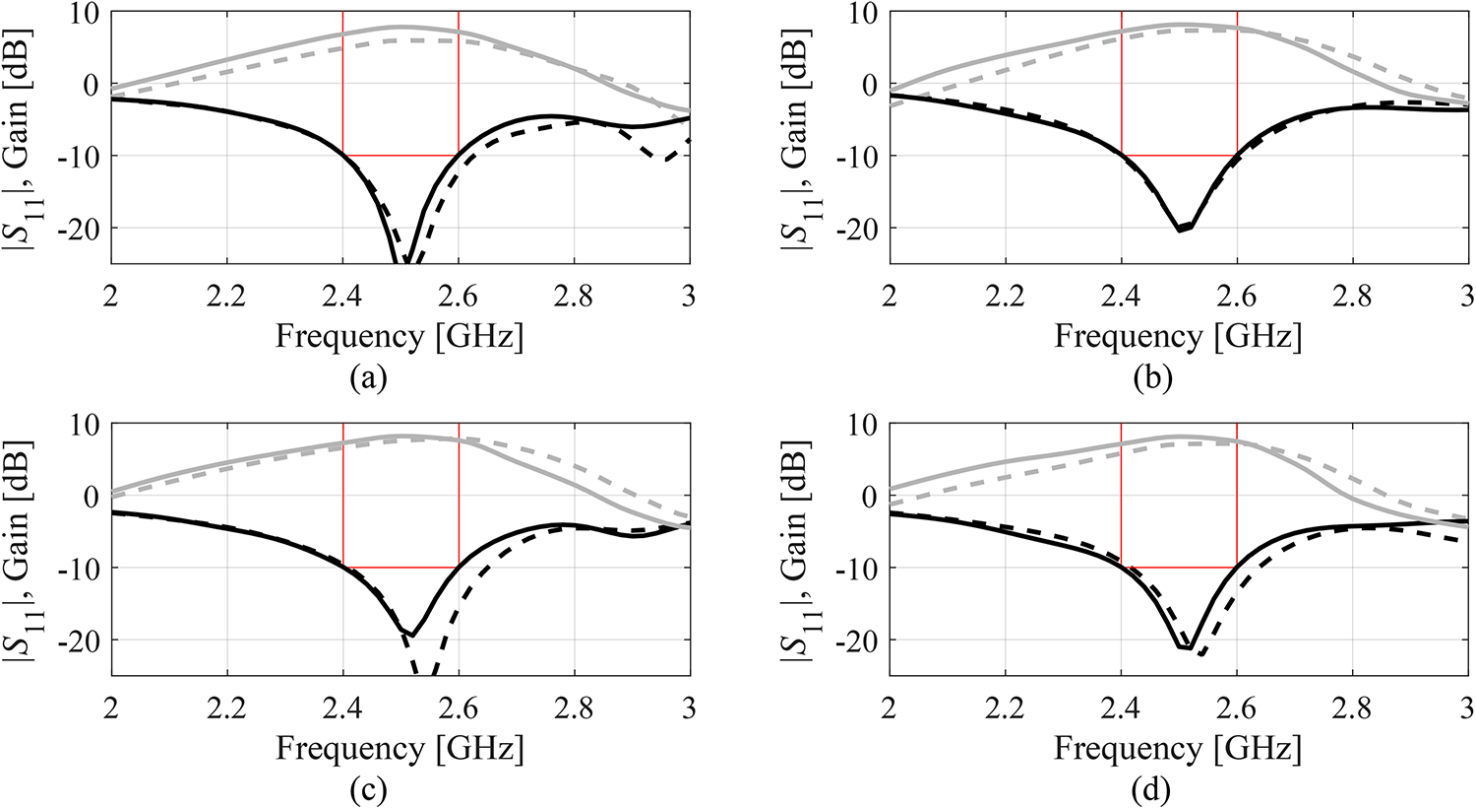
|*S*_11_| and realized gain of Antenna IV at the designs produced by the proposed variable-resolution surrogate-assisted machine learning framework. Shown is the data for selected algorithm runs (plots (**a**) through (**d**)). The design ***x***^(0)^ generated by the global optimization step (- - -) and the final design (—). Target operating bandwidth marked with the horizontal line placed at the acceptance level of − 10 dB for |*S*_11_|.

Finally, Figs. 9, 10, 11, and 12 illustrate antenna characteristics for the designs produced during the selected executions of the suggested methodology. Shown are responses upon concluding the global search stage, and at the final designs.

### Discussion

This section analyzes the results encapsulated in Tables 6, 7, 8, 9 to evaluate the performance of our technique, also in the light of the benchmark procedures. The following paragraphs summarize our findings regarding the major performance indicators, such as design reliability, design quality, and cost-efficiency.

*Optimization process reliability*. The acquired numerical data indicates our method’s perfect success rate (10/10), i.e., its ability to find satisfactory outcomes in each run and for all test cases. Meanwhile, the results obtained for Algorithm IV (gradient search) underscore that the test problems are multimodal indeed. The average success rate for local optimizer is only 4/10. While nature-inspired optimization (PSO, DE, GWO) fares better, it is still not perfect, which confirms insufficiency of the assumed computational budget. Both machine learning frameworks (Algorithms V and VI) perform consistently with 10/10 success rate; however, Algorithm V does not produce designs of the same quality as Algorithm VI and the proposed technique, which is due to operating in the original parameter space, where building of reliable behavioral models is impeded by the dimensionality-related issues. Also, the computational costs of Algorithms V and VI are significantly higher.

*Design quality*. This performance figure is evaluated through the mean cost function value. For Antennas I, II, and III, it is the maximum in-band |*S*_11_|; for Antenna IV it is the end-fire gain at the intended center frequency. The data in Tables 6, 7, 8, 9 shows that the proposed algorithm yields the highest-quality designs, only matched by Algorithm VI. Algorithm V is slightly worse due to operating in the unreduced parameter space and the associated difficulties in surrogate modeling. The quality of results obtained with PSO, DE, and GWO (Algorithms I through III) is noticeably inferior for all antennas, whereas Algorithm IV (gradient search) is clearly inferior due to the fact that—for most runs—local tuning converged to the designs allocated away from the respective optima.

*Computational efficiency*. The expenses incurred by the search process are remarkably low for the presented technique as compared to all global benchmark procedures (Algorithms I, II, III, V, and VI). In terms of average figures, our framework offers over 54-percent savings over Algorithm VI, 72-percent savings over Algorithm V, and about 90-percent savings over Algorithms I, II, and III. The mean running cost is equivalent to less than 140 high-resolution EM simulations, which is in line with that of gradient-based search (~ 110 EM analyses), while offering the global search capability.

*The effects of dimensionality reduction and multi-resolution EM analysis*. The impact of dimensionality reduction and the employment of multi-resolution models becomes evident by comparing the performance of the algorithm of section “Variable-resolution machine learning for global antenna optimization using sensitivity-analysis-based dimensionality reduction” with Algorithms V and VI. Operating in the restricted region *X*_*d*_ leads to a noticeable improvement of the design quality and about forty percent cost reduction. The reason behind both is that limiting the global search stage to *X*_*d*_ improves the accuracy of the surrogate operating within the ML framework and lowers the training data acquisition expenses. The latter is the major contributor to the overall CPU expenses in Algorithm V. On the other hand, utilization of low-resolution EM simulations enabled additional savings (up to the aforementioned 72 percent over Algorithm V). These extra benefits are not detrimental to the design quality. Clearly, the acceleration factor due to variable-resolution modeling is problem dependent as shown in Table 4.

The observations formulated above demonstrate that the proposed variable-resolution machine learning procedure does exhibit global search capability, ensures consistency in terms of producing high-quality designs over multiple algorithm runs and for a variety of design scenarios (optimization of impedance matching, gain enhancement). Its cost efficiency is comparable to local methods, which is perhaps the most essential asset in the context of practical applications. The three fundamental factors contributing to this level of performance include fast sensitivity analysis, dimensionality reduction, and variable-resolution modeling. An additional advantage of the presented technique is that it is easy to set up, which is a consequence of a small number of control parameters.

## Conclusion

The presented study aimed at developing a two-stage methodology for global design optimization of antenna systems. Its keystone components include dimensionality reduction realized by means of fast global sensitivity analysis (FGSA), a machine learning (ML) procedure involving kriging surrogate models, and fine tuning of antenna parameters using accelerated trust-region (TR) search. Restricting the ML process to the FGSA-defined low-dimensionality domain facilitates the construction and refinement of the surrogate model, which is further expedited by conducting this stage using low-resolution EM simulations. On the other hand, the final tuning is carried out in the full-dimensionality space and using high-resolution EM analysis, which ensures the reliability of the optimization process. Extensive verification experiments involved four antennas of diverse responses (multi-band, broadband, enhanced gain). The results demonstrate consistent operation, reliability, repeatability of solutions, and excellent cost efficiency of the presented framework. It is superior to several benchmark approaches that include nature-inspired procedures and machine-learning strategies. The average running expenses of the algorithm correspond to only about 140 EM antenna simulations, which is comparable to the expenses incurred by local optimization. The computational savings due to dimensionality reduction are over fifty percent with the additional advantage being improved design quality. The incorporation of variable-resolution models results in further speedup (over seventy-percent savings over the baseline ML). Other features of the proposed technique include straightforward setup, and a small number of control parameters, due to which there is no need for tuning the procedure to a given optimization problem. Altogether, our framework can be considered an attractive solution approach to antenna optimization tasks, especially when global search capability is necessary and the computational budget is limited.

## Data Availability

The datasets used and/or analyzed during the current study available from the corresponding author on reasonable request.
